# Standard Deviation vs. Gini Coefficient: Effects of Different Indicators of Classroom Status Hierarchy on Bullying Behavior

**DOI:** 10.1007/s10964-024-01956-1

**Published:** 2024-02-24

**Authors:** Sarah T. Malamut, Achiel Fenneman, Claire F. Garandeau

**Affiliations:** 1 INVEST Research Flagship, Department of Psychology, University of Turku, Turku, Finland; 2 SCAN-Unit, Department of Cognition, Emotion, and Methods in Psychology, University of Vienna, Vienna, Austria

**Keywords:** Status hierarchy, Bullying, Adolescence, Classroom context, Popularity

## Abstract

Classroom status hierarchy (the degree to which popularity is unequally distributed in a classroom) has often been examined as a predictor of bullying. Although most research has relied on an operationalization of status hierarchy as the classroom standard deviation (SD) of popularity, other fields (e.g., sociology, economics) have typically measured resource inequality using the Gini coefficient. This multilevel study examines the concurrent and prospective associations of both status hierarchy indicators (referred to as SD-hierarchy and Gini-hierarchy) with peer-reported bullying, controlling for key variables (i.e., the structure of the classroom status hierarchy, average classroom level of popularity). The final sample included 3017 students (45.3% self-identified as a boy; T1 *M*_age_ = 13.04, SD = 1.73, approximately 93% born in Finland) from 209 classrooms. Concurrently, classroom SD-hierarchy was positively, linearly associated with bullying, whereas there was a curvilinear (inverted U) association between Gini-hierarchy and bullying. No significant longitudinal associations were found. The findings suggest that Gini-hierarchy provides unique information beyond the SD-hierarchy.

## Introduction

Classroom status hierarchy, which refers to the degree to which popularity is (un)equally distributed in a classroom, has been shown to be associated with a higher prevalence of bullying in adolescence (Garandeau et al., 2014; Pan et al., 2020). Indeed, peer status is a more limited, and therefore a more valuable, resource in social contexts where a hierarchy is clearly established. Desire for popularity increases in adolescence (LaFontana & Cillessen, 2010) and bullying peers appears to be an effective way to gain (Volk et al., 2012) or maintain (Prinstein & Cillessen, 2003) this resource. Classroom status hierarchy has typically been operationalized as the standard deviation of social status within a classroom or group (e.g., Dawes et al., 2023). However, this operationalization has certain limitations. Knowing the degree to which status is dispersed from the average throughout the classroom does not provide information on the structure of the status hierarchy (e.g., Laninga-Wijnen et al., 2019). In addition, there are other operationalization possibilities that may map more directly onto how status hierarchy is conceptualized, which is inequality of popularity in a classroom. Specifically, in other disciplines (e.g., sociology, economics), inequality of important resources (e.g., wealth) in a context has been measured using the Gini coefficient (Gini, 1921). As the Gini coefficient indicates the degree to which there is perfect equality (Gini = 0) or near perfect inequality (Gini = 1), this operationalization may also be better suited for measuring the degree of popularity (in)equality in a classroom, given that status hierarchy is typically conceptualized as the inequality (rather than dispersion) of status. However, to date, no studies have used the Gini coefficient to operationalize classroom status hierarchy. The current study will fill these gaps by examining the concurrent and prospective associations between two operationalizations of status hierarchy (standard deviation and Gini coefficient) and bullying, while accounting for the structure of the status hierarchy and average classroom level of popularity (as standard deviation is calculated in relation to the mean).

### Status Hierarchy and Bullying: Prior Findings and Limitations

Broadly speaking, two perspectives have been proposed as to how status hierarchies relate to aggression in a group. The *functionalist* perspective posits that a clear, strong status hierarchy deters individuals from being aggressive (Savin-Williams, 1979). Specifically, low-status individuals should realize that it is futile to try to challenge a peer with higher status whereas high-status individuals should realize that their high position in the hierarchy makes it unnecessary for them to aggress against low-status peers. In contrast, the *balance-of-power* perspective argues that a strong status hierarchy enhances the salience and desirability of status as it becomes a more limited and valuable resource (Garandeau et al., 2014). Individuals are then expected to bully more in these contexts, as aggression could be an effective way to gain or maintain this rare resource.

Of the studies that have investigated status hierarchy, some have found a positive association between status hierarchy and bullying or aggression (Garandeau et al., 2014; Pattiselanno et al., 2015) – supporting the *balance-of-power* perspective, whereas others did not find a significant main effect (Babarro et al., 2017; Pan et al., 2020; Zwaan et al., 2013). All of these studies operationalized status hierarchy as the standard deviation of popularity in the classroom. Some focused on concurrent associations (Babarro et al., 2017; Pattiselanno et al., 2015; Zwaan et al., 2013), whereas others investigated longitudinal links (Garandeau et al., 2014; Pan et al., 2020).

**Fig. 1:**
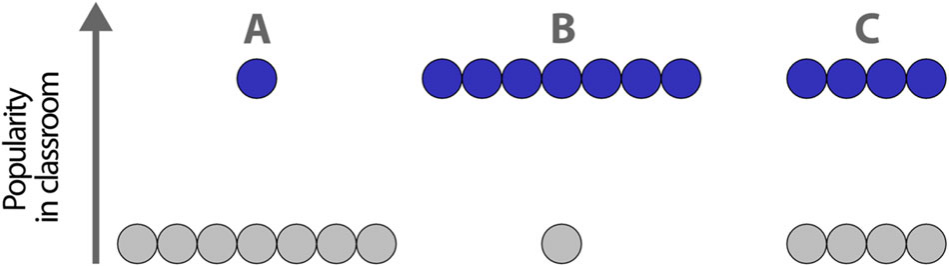
Example of potential configurations of the structure of classroom status hierarchy: **A** pyramid, **B** inverted pyramid, **C** symmetric

This study not only aims to see whether previous findings of a positive concurrent and prospective association between status hierarchy and bullying can be replicated, but also to address important limitations of previous research. Specifically, previous studies did not control for key variables that are likely to influence whether status hierarchy is associated with bullying. First, attention should be paid to the structure of the hierarchy, which refers to the way in which popularity is distributed in each classroom. It has been operationalized as the classroom mean minus the median (Pattiselanno et al., 2015), with positive values indicating few adolescents having high popularity and negative values indicating more highly popular students than unpopular students. It is essential to control for the structure of the hierarchy given that two classrooms could have the same standard deviation of status but different distributions (e.g., Laninga-Wijnen et al., 2019; see Fig. 1A, B). Second, the way popularity is distributed in the classroom may depend on how prevalent popularity is in the classroom. Indeed, the standard deviation of a distribution of scores is calculated based on the mean of the distribution. Therefore, controlling for the average level of popularity might affect the main effect of classroom status hierarchy on bullying.

### A New Operationalization of Classroom Status Hierarchy: The Gini Coefficient

In addition to addressing these limitations, the other main goal of the current study is to examine the Gini coefficient as an alternative way to operationalize status hierarchy. Whereas standard deviation describes the general spread of popularity within a classroom, the Gini coefficient can highlight the extent to which popularity is unequally distributed (e.g., some kids having more popularity while others have less), which more directly taps into how status hierarchy is typically conceptualized. Standard deviation will reach its maximum value if half of the data are at the highest possible value and the other half are at the lowest possible value, whereas the Gini coefficient will reach its maximum value if the data includes one positive value with the rest being zero. That is, standard deviation would reach its maximum if half of the students in the class was very popular and half was very unpopular, whereas the Gini coefficient would reach its maximum if one student was very popular and the rest of the class was not popular at all. Another important point to consider is that there could be scenarios where the standard deviation is low and the structure of the status hierarchy is close to zero but there is still some degree of status inequality in the classroom. Thus, another indicator that is able to capture inequality in these scenarios (e.g., Gini coefficient) is needed.

Previous studies have only considered linear associations between status hierarchy and aggression. Yet, there is reason to expect a curvilinear association, particularly if using the Gini coefficient. The two extreme values of Gini are reached when either everyone is equal (Gini = 0) or one person has popularity when the rest do not have any (Gini = 1); however, bullying may be most prevalent when there are more individuals with some degree of popularity. For example, if there are more students with some levels of popularity (e.g., in the “middle” of the hierarchy), then they may both be driven to *gain* status as they see some peers “above” them in the hierarchy, but also focused on not *losing* status as they see some “below” them in the hierarchy. Consistent with this perspective, a recent study disentangled these two motivations and found that adolescents who were motivated both to avoid low popularity as well as to strive for high popularity were the most aggressive (Lansu & van den Berg, 2023). Thus, when using the Gini coefficient to operationalize status hierarchy (henceforth referred to as “Gini-hierarchy”), a curvilinear association with bullying was hypothesized (specifically an inverted U-shape). A specific hypothesis for curvilinear associations when using the standard deviation to operationalize status hierarchy (henceforth referred to as “SD-hierarchy”) was not posited, but was tested as an exploratory analysis. When testing for the effects of Gini-hierarchy, exploratory models controlling for the average level of popularity in the classroom were also conducted. As with standard deviation, controlling for the average level of popularity could elucidate the extent to which it is specifically inequality of popularity that is related to bullying, rather than the overall level of popularity in the classroom.

## Current Study

Previous studies have highlighted classroom status hierarchy (the degree to which popularity is (un)equally distributed in a classroom) as an important factor related to bullying in the classroom. The first goal of the current study was to replicate previous research that indicated a positive association between SD-hierarchy and bullying, while addressing important limitations of previous research (controlling for the structure of the classroom status hierarchy). The second goal of the current study was to introduce a new way of operationalizing classroom status hierarchy (the Gini coefficient) that may map more directly onto typical conceptualizations of status hierarchy (degree to which popularity is (un)equally distributed). Whereas a linear positive association between SD-hierarchy and bullying was expected based on previous research, the current study tested for a curvilinear association between Gini-hierarchy and bullying. Further addressing the limitations of previous research, the associations were tested with and without controlling for average level of popularity in the classroom, as previous research did not consider the role of the overall popularity in the classroom.

## Method

### Participants and Procedure

The data for this study included Finnish adolescents in grades 4 to 9. Data collection occurred in three waves over the course of one academic year (2020–2021; Wave 1 – October, Wave 2 – February, Wave 3 – May). As this study focused on class status hierarchy, the analyses were conducted on the second and third waves of data (subsequently referred to as T1 and T2) as the status hierarchy may be more established than at the beginning of the school year when some students may not know each other yet. It is important to note that data collection occurred during the COVID-19 pandemic; however, Finland only had brief periods of remote learning, none of which overlapped with the dates of data collection. Active parental consent was obtained from 70.8% of the target sample. Classrooms with fewer than 10 participating students and with participation rates lower than 40% at T1 were excluded from the current analysis, to increase the reliability of peer nomination items (Cillessen & Marks, 2011).

The final sample included 3017 students (45.3% self-identified as a boy; T1 *M*_age_ = 13.04, SD = 1.73, approximately 93% born in Finland) from 209 classrooms, who were present at school the day of data collection. A similar number of students in primary school (grades 4–6; 49.5%) and secondary school (grades 7–9; 50.5%) participated. The majority of students (85.4%) participated in both waves of the data collection. Youth who participated at both times did not significantly differ in bullying from those who only responded at T1 (*p* = 0.32). At T1, less than 2% of data was missing on any variable. Given the low prevalence of missing data, no data imputation was conducted and missing data was handled with listwise deletion. The hypotheses and analytic plan for main analyses were preregistered online at OSF (10.17605/OSF.IO/YS9UG).

## Measures

### Individual-Level Variables

#### Bullying

Bullying was assessed using three items from the Participant Roles Questionnaire (PRQ; i.e., “starts bullying”, “makes the others join in the bullying”, “always finds new ways of harassing the victim”: Salmivali & Voeten, 2004). Participants could nominate an unlimited number of classmates for each item. An individual’s received nominations for each item were summed and divided by the number of possible nominators within each classroom to form proportion scores. Bullying scores were created by averaging the proportion scores across the three items for each student (Cronbach’s αs = 0.92 and 0.91 at T1 and T2).

### Classroom-Level Variables

#### Classroom Status Hierarchy

This study considered both strength and structure of hierarchy. The strength of classroom status hierarchy was assessed in two ways (SD-hierarchy and Gini-hierarchy). First, consistent with previous research (e.g., Garandeau et al., 2014), status hierarchy was calculated by using the standard deviation of popularity for each classroom (SD-hierarchy). Popularity was assessed using peer nominations. Students were asked to nominate their classmates who were the “most popular.” For each participant, the received (unlimited) nominations were summed and divided by the number of possible nominators to form a proportion score, with scores ranging from 0 to 1. SD-hierarchy scores ranged from 0.03 to 0.38 (*M* = 0.14, *SD* = 0.06) across classrooms at T1. The second operationalization of status hierarchy strength was the Gini coefficient of popularity in the classroom (Gini-hierarchy). Gini-hierarchy scores ranged from 0.14 to 1.00 (*M* = 0.62, *SD* = 0.17) at T1.

The structure of classroom status hierarchy was measured by subtracting the median popularity score from the mean popularity score for each classroom. Positive values represent hierarchies where only a few adolescents are popular (pyramid structure), whereas negative values represent classrooms where there are more popular students than unpopular (inverted pyramid structure; Laninga-Wijnen et al., 2019, see Fig. 1 for examples of possible configurations). A score of 0 would represent symmetric classrooms (equal number of popular and unpopular students). Structures of classroom status hierarchy ranged from −0.05 to 0.22 (*M* = 0.06, *SD* = 0.05) at T1.

#### Classroom Size

We calculated the number of participating students in each classroom, and only included classrooms that had at least ten (participating) students.

#### Gender Distribution

We calculated the proportion of boys for each classroom.

#### Classroom Mean Level of Popularity

Participants were asked “Who are the most popular in your class?” and could nominate an unlimited number of classmates. Proportion scores were computed by dividing the number of received nominations by the number of respondents. These scores were then averaged by classroom. The average classroom levels of popularity ranged from 0.01 to 0.31 (*M* = 0.13, *SD* = 0.06) at T1.

### Analytic Plan

A series of multilevel models were performed to examine the concurrent and longitudinal effects of strength of classroom status hierarchy on bullying, using the lme4 package in R (Bates et al., 2015). Separate models were conducted for each indicator of strength of classroom status hierarchy (i.e., SD-hierarchy, Gini-hierarchy). Each model controlled for classroom size, structure of classroom status hierarchy, and the classroom proportion of boys. For each set of analyses, the unconditional means models were tested first and the intraclass correlations (ICCs) were examined. All variables were grand-mean centered (see Enders & Tofighi, 2007). The models were tested with and without controlling for classroom mean level of popularity as an exploratory analysis. The prospective association between classroom status hierarchy and T2 bullying was tested in a model including the T1 classroom-level variables mentioned above (classroom status hierarchy, classroom size, structure of classroom status hierarchy, the proportion of boys within the classroom) as well as controlling for individual-level bullying at T1.

Additional analyses were conducted to test whether there were curvilinear associations between strength of classroom status hierarchy and bullying. In separate models per indicator (SD-hierarchy, Gini-hierarchy), a quadratic term was added to the model for the respective strength of status hierarchy indicator (squaring the uncentered hierarchy indicator), in addition to the linear term. To provide maximum information while avoiding redundancy, unstandardized coefficients are described in text whereas standardized coefficients are presented in the tables.

## Results

### Descriptive Statistics and Intraclass Correlations

Descriptive statistics, stability of individual-level bullying and classroom-level correlations are presented in Table 1. Bullying was highly stable from T1 to T2 (*r* = 0.74). The two indicators of strength of status hierarchy were positively, but only weakly, associated (*r* = 0.07). Also on the classroom level, strength of status hierarchy was positively associated with the structure of the status hierarchy, with stronger associations for SD-hierarchy (*r* = 0.68) than Gini-hierarchy (*r* = 0.32). Whereas classroom mean level of popularity was strongly, positively associated with SD-hierarchy (*r* = 0.70), it was negatively associated with Gini-hierarchy (*r* = −0.59). The intraclass correlations (ICCs) for bullying at T1 and T2 were 0.073 and 0.129, indicating that approximately 7 and 13% of the variance in bullying was due to differences between classrooms at T1 and T2.

**Table 1:** Descriptive Statistics and Correlations of Individual (Level 1) and Classroom-level (Level 2) Variables

	1	2				*M* (SD)
*Individual-level*						
1. Bullying T1	–					0.03 (0.07)
2. Bullying T2	0.74^***^	–				0.02 (0.06)
*Classroom-level*	1	2	3	4	5	
1. SD-hierarchy	–					0.14 (0.06)
2. Gini-hierarchy	0.07^***^	–				0.62 (0.17)
3. Structure of status hierarchy	0.68^***^	0.32^***^	–			0.06 (0.05)
4. Classroom size	−0.12^***^	−0.37^***^	−0.13^***^	–		15.37 (4.11)
5. Classroom proportion of boys	−0.11^***^	−0.00	−0.02	−0.11^***^	–	45.34 (15.33)
6. Mean level of popularity	0.70^***^	−0.59^***^	0.29^***^	0.11^***^	−0.09^***^	0.13 (0.06)

***p < 0.001

**Fig. 2:**
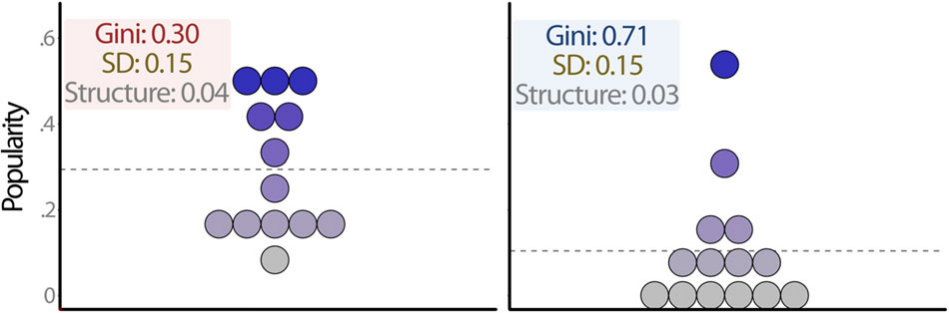
Example of two classrooms with low (left) and high (right) Gini-hierarchy, but near equivalent SD-hierarchy and structure of status hierarchy. The dotted line represents average classroom level of popularity

**Fig. 3:**
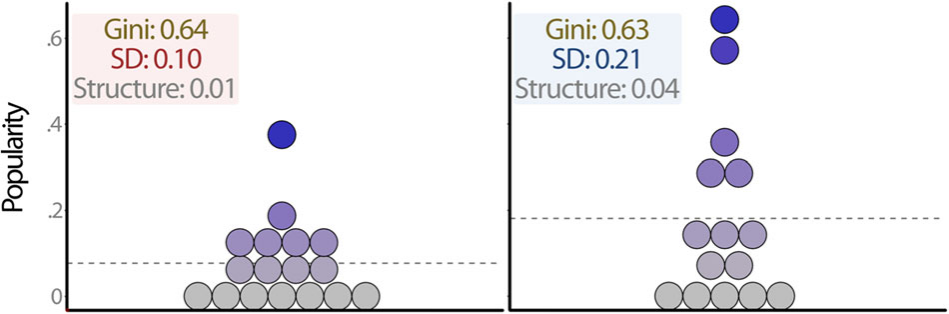
Example of two classrooms with low (left) and high (right) SD-hierarchy, but near equivalent Gini-hierarchy and structure of status hierarchy. The dotted line represents average classroom level of popularity

To further illustrate how the two indicators of status hierarchy (SD-hierarchy, Gini-hierarchy) relate to each other as well as to the structure of the hierarchy, we matched example classrooms in our data based on their scores on these variables. In Fig. 2, we identified two classrooms that had the same standard deviation of popularity (0.15) and almost identical scores for structure of the status hierarchy (0.04 and 0.03), but different Gini coefficients (0.30 vs. 0.71). The hierarchies in these two classrooms still have some key visible differences in their distribution, despite having near equivalent scores on their standard deviation and structure. In Fig. 3, we identified two classrooms that had near equivalent Gini coefficients (0.64 and 0.63), similar structures (0.01 and 0.04), but different standard deviations (0.10 vs. 0.21). The distribution of the hierarchy in these two classrooms are very similar, despite having very different standard deviations. It is, however, important to note that the ranges in Fig. 3 differ, as does the average popularity in these classes. This further underscores the importance of testing the models with and without controlling for average levels of popularity.

### Associations between Status Hierarchy and Bullying

First, the concurrent association between SD-hierarchy and bullying was tested, controlling for classroom size, structure of classroom status hierarchy, and the distribution of gender within the classroom (Table 2, Model 1a). SD-hierarchy was positively associated with bullying (*b* = 0.16, SE = 0.04, *p* < 0.001). Next, a quadratic term for SD-hierarchy was added to test whether there was a curvilinear association (Table 2, Model 1b). However, this was not significant (*p* = 0.70). As a preregistered exploratory analysis, another model was tested while controlling for the classroom mean level of popularity (Table 2, Model 1c) but this model did not include the quadratic term as it was nonsignificant in Model 1b. When controlling for the classroom mean level of popularity, the association between SD-hierarchy (standard deviation) was no longer significant (*b* = 0.10, SE = 0.06, *p* = 0.07).

**Table 2:** Concurrent Associations Between Status Hierarchy T1 and Bullying T1

	Individual-level Bullying T1					
	Status Hierarchy – SD-hierarchy			Status Hierarchy – Gini-hierarchy		
	Model 1a	Model 1b	Model 1c	Model 2a	Model 2b	Model 2c
	*β* (*CI*)	*β* (*CI*)	*β* (*CI)*	*β* (*CI*)	*β* (*CI*)	*β* (*CI)*
Classroom-level variables						
Status hierarchy - linear	0.14^***^ (0.08, 0.21)	0.17^†^ (0.00, 0.35)	0.09^†^ (−0.01, 0.19)	−0.07^*^ (−0.12, −0.01)	0.38^**^ (0.11, 0.65)	0.43^**^ (0.16, 0.69)
Status hierarchy - quadratic	–	−0.03 (−0.21, 0.14)	–	–	−0.45^**^ (−0.72, −0.19)	−0.40^**^ (−0.67, −0.14)
Structure of status hierarchy	−0.08^*^ (−0.15, −0.01)	−0.08^*^ (−0.14, −0.01)	−0.06^†^ (−0.13, 0.01)	0.03 (−0.02, 0.09)	0.02 (−0.03, 0.07)	−0.04 (−0.10, 0.03)
Classroom size	−0.00 (−0.06, 0.05)	−0.00 (−0.06, 0.05)	−0.01 (−0.07, 0.04)	−0.03 (−0.09, 0.03)	−0.04 (−0.10, 0.01)	−0.03 (−0.08, 0.03)
Classroom proportion of boys	0.08^**^ (0.03, 0.13)	0.08^**^ (0.03, 0.13)	0.08^**^ (0.03, 0.13)	0.06^*^(0.01, 0.11)	0.06^*^ (0.01, 0.10)	0.07^**^ (0.02, 0.11)
Mean level of popularity	–	–	0.05 (−0.02, 0.13)	–	–	0.12^**^ (0.04, 0.19)

Standardized coefficients are presented

^†^*p* < 0.10. *p < 0.05. **p < 0.01. ***p < 0.001

**Fig. 4:**
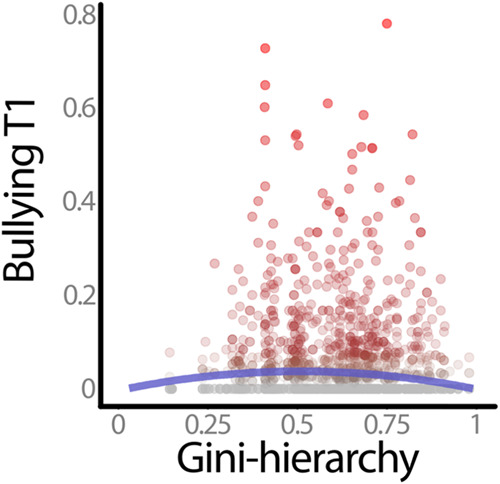
Curvilinear association between Gini-hierarchy and bullying T1. *Note*. The blue line captures the fitted coefficients of both the linear and quadratic terms

Next, the concurrent associations for Gini-hierarchy was tested, still controlling for classroom size, structure of classroom status hierarchy, and the distribution of gender within the classroom (Table 2, Model 2a). There was a negative association between Gini-hierarchy and bullying (*b* = −0.03, SE = 0.01, *p* = 0.02). A quadratic term for the Gini coefficient was then added to test whether there was a curvilinear association (Table 2, Model 2b). The quadratic term was significantly and negatively associated with bullying (*b* = −0.16, SE = 0.05, *p* < 0.001). The graphical depiction of this association (Fig. 4) supported the preregistered hypothesis that there would be an inverted U-shaped curvilinear association, indicating that bullying tended to be higher in classrooms with mid-ranged Gini-hierarchy scores. In Model 2c (Table 2), classroom mean level of popularity was again controlled for as an exploratory analysis. Controlling for mean level of popularity did not affect the curvilinear association depicted in Fig. 4.

After testing the concurrent associations, the prospective associations between status hierarchy at T1 and bullying at T2 were then examined by repeating the models described above while also controlling for bullying at T1. Contrary to expectations, neither SD-hierarchy nor Gini-hierarchy were significant predictors of bullying at T2 in any of the tested models (Table 3).

**Table 3:** Prospective Associations Between Status Hierarchy T1 and Bullying T2

	Individual-level Bullying T2					
	Status Hierarchy – SD-hierarchy			Status Hierarchy – Gini-hierarchy		
	Model 3a	Model 3b	Model 3c	Model 4a	Model 4b	Model 4c
	*β* (*CI*)	*β* (*CI*)	*β* (*CI)*	*β* (*CI*)	*β* (*CI*)	*β* (*CI)*
Individual-level variables						
Bullying T1	0.74^***^ (0.71, 0.76)	0.74^***^ (0.71, 0.76)	0.74^***^ (0.71, 0.76)	0.74^***^ (0.71, 0.76)	0.74^***^ (0.71, 0.76)	0.74^***^ (0.71, 0.76)
Classroom-level variables						
Status hierarchy - linear	−0.02 (−0.08, 0.05)	0.08 (−0.09, 0.25)	−0.04 (−0.14, 0.06)	0.00 (−0.05, 0.05)	0.02 (−0.25, 0.28)	0.00 (−0.08, 0.09)
Status hierarchy - quadratic	–	−0.11 (−0.28, 0.07)	–	–	−0.01 (−0.27, 0.25)	–
Structure of status hierarchy	0.00 (−0.06, 0.07)	0.01 (−0.05, 0.08)	0.01 (−0.06, 0.08)	−0.00 (−0.06, 0.04)	−0.00 (−0.06, 0.04)	−0.00 (−0.07, 0.06)
Classroom size	−0.05^†^ (−0.10, 0.01)	−0.05^†^ (−0.10, 0.00)	−0.05^†^ (−0.11, 0.00)	−0.05 (−0.10, 0.01)	−0.05 (−0.10, 0.01)	−0.05 (−0.10, 0.01)
Classroom proportion of boys	−0.00 (−0.05, 0.04)	−0.00 (−0.05, 0.04)	−0.00 (−0.05, 0.04)	−0.00 (−0.05, 0.04)	−0.00 (−0.05, 0.04)	−0.00 (−0.05, 0.05)
Mean level of popularity	–	–	0.02 (−0.05, 0.10)	–	–	0.00 (−0.07, 0.08)

Standardized coefficients are presented

^†^*p* < 0.10. ***p < 0.001

### Sensitivity Analyses

Given possible developmental and gender differences in bullying behaviors (e.g., López-Castro et al., 2023), a series of sensitivity analyses were conducted, controlling for grade level (classroom-level variable; 0 = primary school, 1 = secondary school) and gender (individual-level variable; 0 = self-identified as a boy, 1 = self-identified as a girl). To ensure that values for gender and grade level added up to 0 (te Grotenhuis et al., 2017), we weighted-effects-coded grade level (secondary school = 0.505 and primary school = −0.495) and gender (girl = 0.51 and boy = −0.49). Given the primary findings regarding the linear vs. quadratic associations between status hierarchy and concurrent bullying, sensitivity analyses were focused on the models with only the linear term for SD-hierarchy and both the linear and quadratic terms for Gini-hierarchy. In most models, grade level and gender were significant predictors such that concurrent levels of bullying were higher for those in primary school and for boys. The main findings of the concurrent models remained the same (Tables S1 and S2), even when controlling for grade level and gender, with one exception. When controlling for grade level and gender, the association between SD-hierarchy and concurrent bullying remained significant even after controlling for classroom mean level of popularity (Table S1, Model S2; *b* = 0.12, SE = 0.06, *p* = 0.04).

Although there were no a priori hypotheses, grade level and gender differences in the associations between classroom hierarchy and bullying were then considered as exploratory analyses. Grade level was not a significant moderator in any of the tested models (Tables S1 and S2). Gender significantly moderated the association between SD-hierarchy and T1 bullying (Table S1, Model S3). In this model, the association between SD-hierarchy and bullying was significant for both boys (*b* = 0.22, SE = 0.04, *p* < 0.001) and girls (*b* = 0.12, SE = 0.04, *p* = 0.01), but was significantly stronger for boys. However, in the model controlling for average classroom level of popularity (Model S4), the association between SD-hierarchy and bullying was still significant for boys (*b* = 0.16, SE = 0.06, *p* = 0.009) but not for girls (*b* = 0.06, SE = 0.06, *p* = 0.37). Gender did not moderate the association between Gini-hierarchy and T1 bullying (Table S2). Controlling for grade level and gender did not impact the findings of the longitudinal analyses, nor were there any significant interactions (Tables S3 and S4).

## Discussion

The classroom context matters for the prevalence of adolescents’ behaviors, such as bullying (Pouwels & Garandeau, 2021). The current study focused on classroom status hierarchy (i.e., the extent to which popularity is (un)equally distributed in the classroom) for several reasons. First, there is strong empirical evidence of positive links between popularity and bullying in adolescence (Wiertsema et al., 2023). Bullying is defined by an imbalance of power, suggesting that contextual characteristics related to inequalities in popularity are important to analyze to better understand the emergence and maintenance of bullying behavior among classmates. Second, some studies have shown that a higher degree of status hierarchy in the classroom contributed to higher rates of bullying (e.g., Garandeau et al., 2014). The current study sought to replicate these findings while addressing limitations of previous studies (i.e., lack of key control variables) as well as introducing a different way to operationalize status hierarchy. Specifically, in addition to using the more traditional operationalization of classroom status hierarchy (the standard deviation of popularity), this study used the Gini coefficient as an indicator of status hierarchy, which is a metric that has been widely used in other disciplines (e.g., sociology, economics) to describe inequalities in the distribution of resources (e.g., Yan & Wen, 2020).

### Differences Between Indicators of Status Hierarchy

The two indicators of status hierarchy were only weakly associated with one another (*r* = 0.07). The example classrooms shown in Figs. 2 and 3 further demonstrate that the two indicators are indeed different ways of capturing status hierarchy. Specifically, the Gini coefficient tapped into differences in the distribution of status within classrooms that would not have been detected using only the standard deviation and the structure of the status hierarchy.

When using SD-hierarchy, support was found for the *balance-of-power* perspective, such that there was more bullying in more hierarchical classrooms, consistent with some previous findings (e.g., Garandeau et al., 2014). This suggests that, in classrooms where popularity scores are more spread out, students tend to score higher in bullying. This association was still present when controlling for the structure of the status hierarchy. Although we tested for curvilinear associations, it was not significant when using the standard deviation.

The *balance-of-power* and *functionalist* perspectives tend to imply linear (but opposing) associations between hierarchy and bullying, when operationalizing status hierarchy as the standard deviation of popularity. However, the curvilinear association when using the Gini-hierarchy suggests additional complexities. As hypothesized, there was a significant, curvilinear (inverted U) association between the Gini-hierarchy and bullying, such that bullying prevalence was highest when there was some degree of status hierarchy (i.e., more students in the “middle”). Bullying was low when there was equality in popularity scores, but bullying was also low in classrooms that were the most unequal based on the Gini coefficient (i.e., in classrooms where one or two students are highly popular and the majority is low in popularity). This finding requires further investigation, as low levels of bullying at high Gini-hierarchy could be explained by both the *functionalist* and *balance-of-power* perspectives. On the one hand, consistent with the *functionalist* approach, most students in such contexts may feel that high status is unachievable and therefore may not even attempt to “bully their way up”. On the other hand, consistent with the *balance-of-power* perspective, students may experience their social environment as relatively egalitarian due to the high number of classmates sharing their same low status, which in turn decreases the visibility or value of status and deters them from engaging in bullying. Thus, additional research is needed to better elucidate how students perceive contexts with different distributions of popularity.

Average classroom levels of popularity were also controlled for as an exploratory analysis. When controlling for average classroom popularity, the linear association between SD-hierarchy and bullying was no longer significant, whereas it did not change the pattern of results when using Gini-hierarchy. Thus, these findings indicate that the Gini coefficient may be more robust to certain control variables. This is not entirely surprising as the standard deviation of popularity in a classroom is calculated based on the average popularity of the classroom. Indeed, in this study, SD-hierarchy was strongly associated with average classroom popularity (*r* = 0.70), whereas the association between Gini-hierarchy and average classroom popularity was strong (but not as strong) yet negative (*r* = −0.59). The strong, positive association between SD-hierarchy and average classroom popularity is not surprising; for example, in classrooms with low levels of popularity, most students will have popularity scores close to zero, whereas classrooms with a higher average popularity will have a larger discrepancy between popular and non-popular classmates. The negative correlation between Gini-hierarchy and average classroom popularity was not predicted, and needs to be replicated given that this is the first study to use the Gini coefficient as an indicator of status hierarchy.

As sensitivity analyses, the models were also conducted while controlling for grade level and gender. Once these variables were controlled for, the association between SD-hierarchy and bullying remained significant, even when controlling for average classroom popularity. Controlling for these variables did not change the association between Gini-hierarchy and bullying. Overall, these findings suggest that the link between SD-hierarchy and bullying may be more affected by key control variables.

In addition to these concurrent associations, prospective associations between status hierarchy and bullying were tested. However, none of the tested associations were significant. The lack of longitudinal findings may be in part due to the high stability of bullying in this sample from T1 to T2 (*β* = 0.74). Moreover, whereas the classroom provides an important context that can shape the prevalence of behaviors, the actual extent to which bullying increases over time is likely due to a multitude of other factors (e.g., individual goals; Caravita & Cillessen, 2012).

### Grade Level and Gender Differences

Separate from the preregistered analyses, exploratory analyses were conducted to test for possible grade level and gender differences in the association between status hierarchy and bullying. There were no significant grade differences in any of the tested models, suggesting that the association between status hierarchy and bullying is similar in primary and secondary schools. However, there were (minimal) gender differences. Without considering average level of popularity, SD-hierarchy was positively associated for both girls and boys, but this association was stronger for boys. With controlling for average level of popularity, this association was only significant for boys. Together, this indicates that the link between status hierarchy and (concurrent) bullying may be stronger for boys, which is consistent with (some) previous findings (Babarro et al., 2017). In general, boys had higher levels of bullying, and this seems to be particularly the case in more hierarchical classrooms. Still, there were no significant gender differences in the association between Gini-hierarchy and bullying, and other previous findings have also not found significant gender differences between status hierarchy and bullying (Pan et al., 2020). Thus, this finding needs further replication and investigation.

### Strengths, Limitations, and Future Directions

The current study rigorously tested whether status hierarchy is associated with bullying by addressing key limitations of previous studies, as well as extending beyond past research by considering a new indicator of status hierarchy (the Gini coefficient). Strengths of this study include a large sample, a new operationalization of status hierarchy, and the inclusion of key control variables.

Still, this study also had some limitations. First, as is the case with most studies on bullying, the bullying variable was skewed. Specifically, a large proportion of students were not viewed by their classmates as engaging in bullying behaviors. Still, as shown in Fig. 4, bullying was highest in classrooms with mid-range Gini-hierarchy despite many cases of individuals who did not bully at all.

Second, the assumed underlying mechanism of why status hierarchy would be related to bullying was not directly tested. Specifically, status hierarchy is presumed to foster an environment in which youth are more likely to use bullying to try to compete for status. However, the current study was unable to test whether hierarchical classrooms actually do increase the feelings of social competition with other classmates. Future research should consider whether adolescents are more likely to engage in social comparisons or feel more competitive for status in hierarchical classrooms.

Third, the association between status hierarchy and bullying likely also depends on the extent to which adolescents feel like they are even able to change their position in the status hierarchy (i.e., the stability of the hierarchy). Adolescents may be more likely to bully in hierarchical classrooms that are dynamic (i.e., more instability), as there would be seemingly more opportunities to climb up (or fall down) the social ladder. Future research should investigate the extent to which status hierarchies are stable, and the potential impact the (in)stability has on the link between status hierarchy and bullying. For example, longitudinal social network analysis could be used to examine changes in bullying behavior for adolescents who maintain their position vs. those who change positions.

Lastly, it should be noted that the Gini coefficient is not an all-encompassing statistic, and its benefits may be maximized when used in conjunction with other parameters (e.g., Blesch et al., 2022) or through the addition of related-but-different operationalizations of inequality (De Maio, 2007). Thus, the point of this study is not to argue that Gini-hierarchy should supplant existing indicators of hierarchy (such as SD-hierarchy, structure of the hierarchy), but instead that it offers an exciting new lens to explain aspects of popularity inequality within classrooms that could not be explained by existing indicators alone. In addition, the Gini-hierarchy offers a new tool to elucidate non-linear association between status hierarchy and adolescents’ social behaviors. Future research could consider how Gini-hierarchy relates to other aspects of the classroom social context, such as bullying norms, friendship centrality or density, antipathies.

## Conclusion

Whereas most previous research has relied on the standard deviation to operationalize status hierarchy in classrooms, the current study built upon this research by considering an alternative indicator of status hierarchy – the Gini coefficient. It demonstrated that the classroom standard deviation of popularity and the Gini coefficient are not equivalent measures of classroom status hierarchy. The findings replicated previous findings of a positive, linear association between SD-hierarchy and bullying; however, this finding no longer reached significance when accounting for other classroom characteristics (classroom average level of popularity). Crucially, the findings with the Gini coefficient suggest additional complexity between status hierarchy and bullying behavior, as there was a non-linear (inverted-U) association between Gini-hierarchy and bullying. This study shows that the measurement of status hierarchy may benefit from the use of multiple indicators and underscores the importance of carefully considering control variables.

## Supplementary information

Supplementary Information

## References

[CR1] Barbarro, M. J., Díaz-Aguado, M. J., Arias, R. M., & Steglich, C. (2017). Power structure in the peer group: The role of classroom cohesion and hierarchy in peer acceptance and rejection of victimized and aggressive students. *Journal of Early Adolescence*, *37*, 1197–1220. 10.1177/0272431616648451.

[CR2] Bates, D., Mächler, M., Bolker, B., & Walker, S. (2015). Fitting linear mixed-effects models using lme4. *Journal of Statistical Software*, *67*(1), 48 10.18637/jss.v067.i01.

[CR3] Blesch, K., Hauser, O. P., & Jachimowicz, J. M. (2022). Measuring inequality beyond the Gini coefficient may clarify conflicting findings. *Nature Human Behaviour*, *6*, 1525–1536. 10.1038/s41562-022-01430-7. 10.1038/s41562-022-01430-7 PMC7614289 36038775

[CR4] Caravita, S. C. S., & Cillessen, A. H. N. (2012). Agentic or communal? Associations between interpersonal goals, popularity, and bullying in middle childhood and early adolescence. *Social Development*, *21*, 376–395. 10.1111/j.1467-9507.2011.00632.x.

[CR5] Cillessen, A. H., & Marks, P. E. (2011). Conceptualizing and measuring popularity. In A. H. N. Cillessen, D. Schwartz, & L. Mayeux (Eds.), *Popularity in the peer system* (pp. 25–56). Guilford Press.

[CR6] Dawes, M., Starrett, A., Norwalk, K., Hamm, J., & Farmer, T. (2023). Student, classroom, and teacher factors associated with teachers’ attunement to bullies and victims. *Social Development*, *32*, 922–943. 10.1111/sode.12669.

[CR7] De Maio, F. G. (2007). Income inequality measures. *Journal of Epidemiology and Community Health*, *61*, 849–852. 17873219 10.1136/jech.2006.052969 PMC2652960

[CR8] Enders, C. K., & Tofighi, D. (2007). Centering predictor variables in crosssectional multilevel models: A new look at an old issue. *Psychological Methods*, *12*, 121–138. 10.1037/1082-989X.12.2.121. 17563168 10.1037/1082-989X.12.2.121

[CR9] Garandeau, C., Lee, I., & Salmivalli, C. (2014). Inequality matters: classroom status hierarchy and adolescents’ bullying. *Journal of Youth and Adolescence*, *43*, 1123–1133. 10.1007/s10964-013-0040-4. 24129884 10.1007/s10964-013-0040-4

[CR10] Gini, C. (1921). Measurement of inequality of incomes. *The Economic Journal*, *31*, 124–125.

[CR11] LaFontana, K. M., & Cillessen, A. H. N. (2010). Developmental changes in the priority of perceived status in childhood and adolescence. *Social Development*, *19*, 130–147. 10.1111/j.1467-9507.2008.00522.x.

[CR12] Laninga‐Wijnen, L., Harakeh, Z., Garandeau, C. F., Dijkstra, J. K., Veenstra, R., & Vollebergh, W. A. (2019). Classroom popularity hierarchy predicts prosocial and aggressive popularity norms across the school year. *Child Development*, *90*, e637–e653. 10.1111/cdev.13228. 30825397 10.1111/cdev.13228 PMC6849822

[CR13] Lansu, T., & van den Berg, Y. (2023). *Being on top versus not dangling at the bottom: Popularity motivation and aggression in youth*. PsyArXiv. 10.31234/osf.io/zrmve. 10.1002/ab.22163 38949228

[CR14] López-Castro, L., Smith, P. K., Robinson, S., & Görzig, A. (2023). Age differences in bullying victimisation and perpetration: Evidence from cross-cultural surveys. *Aggression and Violent Behavior*, *73*, 101888. 10.1016/j.avb.2023.101888.

[CR15] Pan, B., Zhang, L., Ji, L., Garandeau, C. F., Salmivalli, C., & Zhang, W. (2020). Classroom status hierarchy moderates the association between social dominance goals and bullying behavior in middle childhood and early adolescence. *Journal of Youth and Adolescence*, *49*, 2285–2297. 10.1007/s10964-020-01285-z. 32661845 10.1007/s10964-020-01285-z

[CR16] Pattiselanno, K., Dijkstra, J. K., Steglich, C., Vollebergh, W. A. M., & Veenstra, R. (2015). Structure matters: The role of clique hierarchy in the relationship between adolescent social status and aggression and prosociality. *Journal of Youth and Adolescence*, *44*, 2257–2274. 10.1007/s10964-015-0310-4. 26077559 10.1007/s10964-015-0310-4 PMC4636991

[CR17] Pellegrini, A. D., & Long, J. D. (2002). A longitudinal study of bullying, dominance, and victimization during the transition from primary school through secondary school. *British Journal of Developmental Psychology*, *20*, 259–280. 10.1348/026151002166442.

[CR18] Pouwels, J. L., & Garandeau, C. F. (2021). The role of the peer group and classroom factors in bullying behavior. In Smith, P. K., & O’Higgins Norman, J. (Eds.), *The Wiley Blackwell handbook of bullying: A comprehensive and international review of research and intervention* (Vol. 1, pp. 450-). Wiley-Blackwell.

[CR19] Prinstein, M. J., & Cillessen, A. H. (2003). Forms and functions of adolescent peer aggression associated with high levels of peer status. *Merrill-Palmer Quarterly*, *49,* 310–342.

[CR20] Salmivalli, C., & Voeten, M. (2004). Connections between attitudes, group norms, and behaviour in bullying situations. *International Journal of Behavioral Development*, *28*, 246–258. 10.1080/01650250344000488.

[CR21] Savin-Williams, R. C. (1979). Dominance hierarchies in groups of early adolescents. *Child Development*, *50*, 923–935. 10.2307/1129316.

[CR22] te Grotenhuis, M., Pelzer, B., Eisinga, R., Nieuwenhuis, R., Schmidt-Catran, A., & Konig, R. (2017). When size matters: advantages of weighted effect coding in observational studies. *International Journal of Public Health*, *62*, 163–167. 10.1007/s00038-016-0901-1. 27796415 10.1007/s00038-016-0901-1 PMC5288425

[CR23] Volk, A. A., Camilleri, J. A., Dane, A. V., & Marini, Z. A. (2012). Is adolescent bullying an evolutionary adaptation? *Aggressive Behavior*, *38*, 222–238. 10.1007/s42380-018-0005-y. 22331629 10.1002/ab.21418

[CR24] Wiertsema, M., Vrijen, C., van der Ploeg, R., Sentse, M., & Kretschmer, T. (2023). Bullying perpetration and social status in the peer group: A meta-analysis. *Journal of Adolescence*, *95*(1), 34–55. 10.1002/jad.12109. 36281722 10.1002/jad.12109 PMC10092515

[CR25] Yan, B., & Wen, B. (2020). Income inequality, corruption and subjective well-being. *Applied Economics*, *52*, 1311–1326.

[CR26] Zwaan, M., Dijkstra, J. K., & Veenstra, R. (2013). Status hierarchy, attractiveness hierarchy and sex ratio. *International Journal of Behavioral Development*, *37*, 211–221. 10.1177/0165025412471018.

