# Correction: Standard Deviation vs. Gini Coefficient: Effects of Different Indicators of Classroom Status Hierarchy on Bullying Behavior

**DOI:** 10.1007/s10964-024-02000-y

**Published:** 2024-05-29

**Authors:** Sarah T. Malamut, Achiel Fenneman, Claire F. Garandeau

**Affiliations:** 1 INVEST Research Flagship, Department of Psychology, University of Turku, Turku, Finland; 2 SCAN-Unit, Department of Cognition, Emotion, and Methods in Psychology, University of Vienna, Vienna, Austria

Correction to: Journal of Youth and Adolescence (2024)

10.1007/s10964-024-01956-1

In the original publication of the article, the author has missed to include the following sentence “This research (including data collection) was also supported by the Challenge Project (ERC-AdG 2019: 884434; awarded to Prof. Christina Salmivalli) which should be included as a second sentence in Acknowledgement section. This has now been corrected with this erratum.

**Acknowledgements** This research was partially supported by the INVEST Research Flagship Centre, funded by the Research Council of Finland (decision number: 345546). This research (including data collection) was also supported by the Challenge Project (ERC-AdG 2019: 884434; awarded to Prof. Christina Salmivalli). Correspondence concerning this article should be addressed to Sarah Malamut, INVEST Research Flagship, Department of Psychology, University of Turku, 20500 Turku, Finland. Fully anonymized data and analysis code are available from the first author upon reasonable request. Electronic mail may be sent to sarah.malamut@utu.fi

