# Supplementary material for: Standard Deviation vs. Gini Coefficient: Effects of Different Indicators of Classroom Status Hierarchy on Bullying Behavior
Source: J Youth Adolesc. 2024 Feb 24;53(8):1875–85. doi: 10.1007/s10964-024-01956-1 (PMC11226515; doi:10.1007/s10964-024-01956-1)
Supplement: Supplementary file 1 — Supplementary Information [file 10964_2024_1956_MOESM1_ESM.docx]

Table S1
*Concurrent Associations Between SD-Hierarchy T1 and Bullying T1*

|  | Individual-level Bullying T1 | | | |
| --- | --- | --- | --- | --- |
|  | Model S1 | Model S2 | Model S3 | Model S4 |
|  | *β* (*CI*) | *β* (*CI*) | *β* (*CI*) | *β* (*CI)* |
| Individual-level variables |  |  |  |  |
| Gender | -.49^***^  *(-.56, -.42)* | -.49^***^  *(-.56, -.42)* | -.49^***^  *(-.56, -.42)* | -.49^***^  *(-.56, -.42)* |
| Classroom-level variables |  |  |  |  |
| SD-hierarchy | .15^***^ (*.09, .22*) | .10^*^ *(.00, .20)* | .20^***^ (*.12, .28*) | .14^**^ (*.04, .25*) |
| Structure of status hierarchy | -.08^*^ (-*.15, -.01*) | -.06† *(-.13, .01*) | -.08^*^ (-*.14, -.01*) | -.06 (-*.13, .01*) |
| Classroom size | -.02  *(-.07, .04)* | -.03 (*-.08, .03)* | -.02  *(-.07, .04)* | -.03  *(-.09, .03)* |
| Classroom proportion of boys | .01 (-*.04, .06*) | .01 (*-.04, .06)* | .00 (-*.05, .05*) | .00 (-*.05, .05*) |
| Grade level | -.08^**^ *(-.13, -.03)* | -.08^**^ *(-.13, -.03)* | -.08^**^ *(-.13, -.03)* | -.08^**^ *(-.13, -.03)* |
| Mean level of popularity | -- | .05 (-.02, .13) | -- | .05 (-*.02, .13*) |
| Gender X SD-hierarchy | -- |  | -.10^**^ *(-.17, -.02)* | -.10^**^ *(-.17, -.02)* |
| Grade X SD-hierarchy | -- |  | .01 *(-.04, .06)* | .01 *(-.04, .06)* |

*Note*. Standardized coefficients are presented. Weighted-effects-codes of self-identified gender: Girls = 0.51 and boys = −0.49. Weighted-effects-codes of grade level: Secondary school = .505 and primary school = -.495.

†*p* <.10. **p* < .05. ***p* < .01. ****p* < .001.

Table S2
*Concurrent Associations Between Gini-Hierarchy T1 and Bullying T1*

|  | Individual-level Bullying T1 | | | |
| --- | --- | --- | --- | --- |
|  | Model S5 | Model S6 | Model S7 | Model S8 |
|  | *β* (*CI*) | *β* (*CI*) | *β* (*CI*) | *β* (*CI)* |
| Individual-level variables |  |  |  |  |
| Gender | -.49^***^ (-*.56, -.41*) | -.49^***^ (-*.56, -.42*) | -.35^*^ (-*.63, -.07*) | -.34^*^ (-*.62, -.07*) |
| Classroom-level variables |  |  |  |  |
| Gini-hierarchy - linear | .32^*^ (*.05, .60*) | .38^**^ (*.11, .65*) | 2.86^*^ (*.49, 5.23*) | 2.60^*^ *(.27, 4.94)* |
| Gini-hierarchy - quadratic | -.39^**^ (-*.66, -.12*) | -.33^*^ (-*.60, -.07*) | -2.96^*^ (-*5.33, -.58*) | -2.59^*^ (*-4.93, -.24*) |
| Structure of status hierarchy | .03 (-*.02, .08*) | .04 (-*.11, .02*) | .05† (-*.01, .10*) | -.03 (-*.09, .04*) |
| Classroom size | -.05†  *(-.11, .00)* | -.03  *(-.09, .02)* | -.07^*^  *(-.13, -.01)* | -.05 *(-.10, .01)* |
| Classroom proportion of boys | -.02 (-*.07, .03*) | -.00 (-*.05, .05*) | -.02 (-*.07, .03*) | -.01 (-*.06, .04*) |
| Grade level | -.07^**^ (-*.12, -.02*) | -.07^**^ (-*.12, -.02*) | .15 (-*.15, .45*) | .15 (-*.14, .44*) |
| Mean level of popularity | -- | .14^***^ (*.07, .22*) | -- | .13^**^ (*.05, .21*) |
| Gini-hierarchy (linear) X Gini-hierarchy (quadratic) | -- |  | .34† (*.00, .68*) | .29† (-*.05, .63*) |
| Gender X Gini-hierarchy (linear) | -- |  | -1.42 (*-3.25, .42*) | -1.45 (*-3.29, .38*) |
| Gender X Gini-hierarchy (quadratic) | -- |  | 1.46 (*-.39, 3.31*) | 1.50 (*-.35, 3.35*) |
| Gender X Gini-hierarchy (linear) X Gini-hierarchy (quadratic) | -- |  | -.14 (*-.41, .13*) | -.15 (*-.42, .13*) |
| Grade X Gini-hierarchy (linear) | -- |  | -1.42 (*-3.53, .69*) | -1.45 (*-3.51, .61*) |
| Grade X Gini-hierarchy (quadratic) | -- |  | 1.45 (*-.66, 3.56*) | 1.47 (*-.59, 3.53*) |
| Grade X Gini-hierarchy (linear) X Gini-hierarchy (quadratic) | -- |  | -.23 (*-.52, .07*) | -.23 (*-.52, .06*) |

*Note*. Standardized coefficients are presented. Weighted-effects-codes of self-identified gender: Girls = 0.51 and boys = −0.49. Weighted-effects-codes of grade level: Secondary school = .505 and primary school = -.495.

†*p* <.10. **p* < .05. ***p* < .01. ****p* < .001.

Table S3
*Prospective Associations Between SD-Hierarchy T1 and Bullying T2*

|  | Individual-level Bullying T2 | | | |
| --- | --- | --- | --- | --- |
|  | Model S9 | Model S10 | Model S11 | Model S12 |
|  | *β* (*CI*) | *β* (*CI*) | *β* (*CI*) | *β* (*CI)* |
| Individual-level variables |  |  |  |  |
| Bullying T1 | .73^***^ (*.70, .76*) | .73^***^ (*.70, .76*) | .73^***^ (*.70, .76*) | .73^***^ (*.70, .76*) |
| Gender | -.05† (-*.11, .00*) | -.05† (-*.11, .00*) | -.05† (-*.11, .00*) | -.05† (-*.11, .00*) |
| Classroom-level variables |  |  |  |  |
| SD-hierarchy | -.02 (-*.09, .05*) | -.05 (-*.15, .05*) | -.04 (-*.11, .04*) | -.07 *(-.17, .04)* |
| Structure of status hierarchy | .01 (-*.06, .08*) | .02 (-*.05, .10*) | .01 (-*.06, .08*) | .02 (-*.05, .10*) |
| Classroom size | -.05†  (*-.11, .01*) | -.06†  (*-.11, .00*) | -.05†  (*-.11, .01*) | -.06† *(-.11, .00)* |
| Classroom proportion of boys | -.01 (-*.06, .04*) | -.01 (-*.06, .04*) | -.01 (*-.06, .04*) | -.01 (-*.06, .04*) |
| Grade level | -.01 (-*.06, .05*) | -.00 (-*.06, .05*) | -.01 (-*.06, .05*) | -.01 (-*.06, .05*) |
| Mean level of popularity | -- | .03 (-*.04, .11*) | -- | .05 (-*.02, .13*) |
| Gender X SD-hierarchy | -- |  | .03 (-*.02, .08*) | .03 (-*.02, .08*) |
| Grade X SD-hierarchy | -- |  | -.00 (-*.05, .05*) | -.00 (-*.02, .08*) |

*Note*. Standardized coefficients are presented. Weighted-effects-codes of self-identified gender: Girls = 0.51 and boys = −0.49. Weighted-effects-codes of grade level: Secondary school = .505 and primary school = -.495.

†*p* <.10. **p* < .05. ***p* < .01. ****p* < .001.

Table S4
*Prospective Associations Between Gini-Hierarchy T1 and Bullying T2*

|  | Individual-level Bullying T2 | | | |
| --- | --- | --- | --- | --- |
|  | Model S13 | Model S14 | Model S15 | Model S16 |
|  | *β* (*CI*) | *β* (*CI*) | *β* (*CI*) | *β* (*CI)* |
| Individual-level variables |  |  |  |  |
| Bullying T1 | .73^***^ (*.70, .76*) | .73^***^ (*.70, .76*) | .73^***^ (*.70, .76*) | .73^***^ (*.70, .76*) |
| Gender | -.05† (-*.11, .00*) | -.05† (-*.11, .00*) | -.00 (-*.21, .21*) | -.02 (-*.29, .33*) |
| Classroom-level variables |  |  |  |  |
| Gini-hierarchy - linear | -.01  (-*.28, .26*) | -.01  (-*.29, .26*) | .39 (*-1.89, 2.68*) | .40 *(-.1.90, 2.69)* |
| Gini-hierarchy - quadratic | .01  (-*.26, .28*) | .01  (-*.27, .28*) | -.40  (*-2.68, 1.88*) | -.40 *(-.2.70, 1.89)* |
| Structure of status hierarchy | .00 (-*.05, .05*) | .00 (-*.07, .07*) | -.00 (-*.06, .05*) | .00 (-*.07, .07*) |
| Classroom size | -.05†  (*-.11, .01*) | -.05†  (*-.11, .01*) | -.05  (*-.11, .01*) | -.05 *(-.11, .01)* |
| Classroom proportion of boys | -0.01 (-*.06, .04*) | -0.01 (-*.06, .04*) | .01 (-*.06, .04*) | -.01 (-*.06, .04*) |
| Grade level | -0.00 (-*.06, .05*) | -0.00 (-*.06, .05*) | -.02 (-*.29, .33*) | -.02 (-*.29, .33*) |
| Mean level of popularity | -- | -0.00 (-*.08, .08*) | -- | -.00 (-*.08, .08*) |
| Gini-hierarchy (linear) X Gini-hierarchy (quadratic) | -- |  | -.08 (-*.25, .41*) | -.08 (-*.25, .41*) |
| Gender X Gini-hierarchy (linear) | -- |  | -.29 (*-1.65, 1.08*) | -.29 (*-1.65, 1.08*) |
| Gender X Gini-hierarchy (quadratic) | -- |  | .29 (*-1.08, 1.67*) | .29 (*-1.08, 1.67*) |
| Gender X Gini-hierarchy (linear) X Gini-hierarchy (quadratic) | -- |  | -.06 (*-.26, .15*) | -.06 (*-.26, .15*) |
| Grade X Status hierarchy (linear) | -- |  | -.08 (*-2.27, 2.11*) | -.08 (*-2.28, 2.12*) |
| Grade X Gini-hierarchy (quadratic) | -- |  | .06 (*-2.12, 2.25*) | .06 (*-2.13, 2.25*) |
| Grade X Gini-hierarchy (linear) X Gini-hierarchy (quadratic) | -- |  | -.03 (-*.34, .28*) | -.03 (-*.34, .28*) |

*Note*. Standardized coefficients are presented. Weighted-effects-codes of self-identified gender: Girls = 0.51 and boys = −0.49. Weighted-effects-codes of grade level: Secondary school = .505 and primary school = -.495.

†*p* <.10. **p* < .05. ***p* < .01. ****p* < .001.
